# Results after surgical treatment of periprosthetic proximal femoral fractures. Osteosynthesis with prosthesis preservation vs. prosthesis change

**DOI:** 10.3205/iprs000146

**Published:** 2020-09-18

**Authors:** Dirk Zajonz, Cathleen Pönick, Melanie Edel, Robert Möbius, Christian Pfeifle, Torsten Prietzel, Andreas Roth, Johannes K. M. Fakler

**Affiliations:** 1 Department of Orthopaedic Surgery, Traumatology and Plastic Surgery, University Hospital Leipzig, Leipzig, Germany; 2 ZESBO – Center for research on musculoskeletal systems, Leipzig, Germany; 3 Clinic for Orthopaedics, Trauma and Reconstructive Surgery, Zeisigwald Hospital Bethania, Chemnitz, Germany

**Keywords:** periprosthetic proximal femoral fracture, osteosynthesis, prosthesis change

## Abstract

**Background:** Periprosthetic fractures (PPF) of the femur close to the hip joint have serious consequences for most geriatric affected patients. In principle, apart from the highly uncommon conservative therapy, there are two therapeutic options. On the one hand, the prosthesis-preserving treatment by means of osteosynthesis using plates and/or cerclages in general is available. On the other hand, a (partial) change of the prosthesis with optionally additive osteosynthesis or a proximal femoral replacement can be performed because of prosthesis loosening or non-reconstructable comminuted fractures as well as most cemented stem variations.

The aim of this retrospective study is the analysis of periprosthetic proximal femoral fractures in the presence of a total hip arthroplasty (THA). The outcome of the operated patients is to be investigated depending on the type of care (osteosynthesis with prosthesis preservation vs. prosthesis change).

**Material and methods:** In a retrospective case analysis, 80 patients with THA and PPF were included. They were divided into two groups. Group I represents the osteosynthetic treatment to preserve the implanted THA (n=42). Group II (n=38) includes those patients who were treated by a change of their endoprosthesis with or without additional osteosynthesis. Specifics of all patients, like gender, age at fracture, interval between fracture and implantation, length of in-patient stay, body mass index, osteoporosis, corticomedullary index and complications such as infections, re-fracture, loosening, material failure or other complications, were recorded and compared. Furthermore, the patients were re-examined by a questionnaire and the score according to Merle d’Aubigné and Postel.

**Results:** In group I the mean follow-up time was 48.5±23 months (4 years) whereas group II amounted 32.5±24.5 months (2.7 years) (p=0.029). Besides, there were significant differences in age (81± 11 years vs. 76±10 years, p=0.047) and length of in-patient stay (14.5±8.6 days vs. 18.0±16.7 days, p=0.014). According to the score of Merle d’Aubigné and Postel, there were significantly better values for the pain in group II with comparable values for mobility and walking ability.

**Conclusion:** The treatment of periprosthetic proximal fractures of the femur is dependent on the classification (Vancouver and Johannsen) and in particular on the prosthetic anchoring as well as the extent of the comminution zone. Older patients and patients with osteoporosis are more frequently treated with an endoprosthesis revision. Patients, who have been treated with an osteosynthesis for preserving their endoprosthesis, showed a shorter length of in-patient stay and fewer complications than people with replacement surgery. In contrast to that, patients with prosthesis revision had better outcomes concerning the score of Merle d’Aubigné and Postel.

## Zusammenfassung

**Hintergrund: **Periprothetische Frakturen (PPF) des hüftgelenksnahen Oberschenkelknochens haben für die meist geriatrischen Patienten schwerwiegende Folgen. Prinzipiell gibt es neben der sehr seltenen konservativen Therapie zwei therapeutische Optionen. Zum einen ist dies das prothesenerhaltende Vorgehen mittels Osteosynthese durch Platten und/oder Cerclagen. Zum anderen kann aufgrund von Prothesenlockerungen oder nicht rekonstruierbaren Trümmerfrakturen sowie den meisten zementierten Schaftvariationen ein (teilweiser) Prothesenwechsel mit wahlweise additiver Osteosynthese oder ein proximaler Femurersatz durchgeführt werden.

Das Ziel dieser retrospektiven Studie ist die Analyse von periprothetischen proximalen Femurfrakturen bei einliegender Hüfttotalendoprothese. Je nach Art der Versorgung (Osteosynthese mit Endoprothesenerhalt gegenüber Endoprothesenwechsel) soll das Ergebnis der operierten Patienten untersucht werden.

**Material und Methoden:** In einer retrospektiven Fallanalyse wurden 80 Patienten mit HTEP und PPF eingeschlossen. Sie wurden in zwei Gruppen eingeteilt. Gruppe I repräsentiert die osteosynthetische Behandlung zum Erhalt der implantierten HTEP (n=42). Gruppe II (n=38) umfasst die Patienten, die durch einen Endoprothesenwechsel mit oder ohne zusätzliche Osteosynthese behandelt wurden. Die Besonderheiten aller Patienten, wie Geschlecht, Alter bei Fraktur, Intervall zwischen Fraktur und Implantation, Dauer des stationären Aufenthalts, Body-Mass-Index, Osteoporose, kortikomedullärer Index und Komplikationen wie Infektionen, Re-Fraktur, Lockerung, Materialversagen oder andere Komplikationen, wurden erfasst und verglichen. Darüber hinaus wurden die Patienten anhand eines Fragebogens erneut untersucht und der Score nach Merle d'Aubigné und Postel ermittelt.

**Ergebnisse:** In Gruppe I betrug die mittlere Nachbeobachtungszeit 48,5±23 Monate (4 Jahre), während sie in Gruppe II 32,5±24,5 Monate (2,7 Jahre) betrug (p=0,029). Außerdem gab es signifikante Unterschiede im Alter (81±11 Jahre vs. 76±10 Jahre, p=0,047) und in der Dauer des stationären Aufenthalts (14,5±8,6 Tage vs. 18,0±16,7 Tage, p=0,014). Gemäß dem Score von Merle d‘Aubigné und Postel ergaben sich signifikant bessere Werte für die Schmerzen in Gruppe II mit vergleichbaren Werten für Mobilität und Gehfähigkeit.

**Schlussfolgerung:** Die Behandlung von periprothetischen proximalen Femurfrakturen ist abhängig von der Klassifikation (Vancouver und Johannsen) und insbesondere von der prothetischen Verankerung sowie der Ausdehnung der Trümmerzone. Ältere Patienten und Patienten mit Osteoporose werden häufiger mit einer Endoprothesenrevision behandelt. Patienten, die unter Erhaltung der Endoprothese mit einer Osteosynthese behandelt wurden, zeigten eine kürzere stationäre Verweildauer und weniger Komplikationen als Patienten mit einer Wechseloperation. Im Gegensatz dazu hatten Patienten mit Prothesenrevision bessere Ergebnisse hinsichtlich des Scores von Merle d‘Aubigné und Postel.

## Introduction

The increasing number of primary total hip endoprosthesis (THA) with longer implant lifetimes as well as associated age- and implant-related changes due to osteoporosis and wear reactions is leading to an accumulation of revisions based on complications [1], [2], [3], [4]. A nationwide survey of total hip arthroplasty (THA) revisions in the USA showed an increase of 39% in THA replacements of 235,857 analyzed cases between 2005 and 2010. Luxations (22%) and mechanical loosening (20%) were the most common causes of replacement surgery [1]. Osteolysis as a result of polyethylene debris is discussed as a major cause of implant loosening [5], [6]. In addition to this endoprosthetic loosening, the reduced bone quality due to osteoporosis and the physical impairment of mostly geriatric patients with a tendency to fall lead to an increased periprosthetic fracture risk [7], [8]. The frequency of periprosthetic fractures (PPF) after THA varies between 0.1 and 4.5%, depending on age, gender, implant life, anchorage and associated diseases, and increases to 3.6 to 20.9% after implant revision [9], [10]. PPF have serious consequences for most geriatric affected patients. In particular, a long immobilization increases the risk of nosocomial infections, thromboses as well as embolisms and thus the mortality [11]. Due to many existing dislocations, the lack of primary bone healing, the doubtful stability of the prosthesis and the long-lasting relief, conservative treatment is possible only in individual cases but generally not indicated [12]. The main treatment goals are a gentle stabilization with restitution of joint function and an early load-bearing capacity of the affected limb in order to ensure rapid postoperative mobilization of the respective patients. In principle, apart from the highly uncommon conservative therapy, there are two therapeutic options. On the one hand, the prosthesis-preserving treatment by means of osteosynthesis using plates and/or cerclages in general is available [9], [13]. This is indicated with appropriate fracture morphology and firm anchoring of the prosthetic stem in the femur. In the case of prosthesis loosening or non-reconstructable comminuted fractures as well as in the case of most cemented stem variations, a prosthesis (partial) change with additive osteosynthesis if necessary up to a proximal femoral replacement is indispensable [11], [14]. Ultimately, the choice of the appropriate treatment method is multifactorial and also dependent on the experience of the surgeon. Often it can be definitively determined only during surgery [14].

The aim of this retrospective study is to analyze periprosthetic proximal femoral fractures of patients with THA treated in our clinic. Finally, the outcome of the operated patients is to be investigated depending on the type of care (osteosynthesis with prosthesis preservation vs. prosthesis change).

## Material and methods

Before conducting this study, the vote of our university ethics committee was obtained (044/14032016).

A retrospective case analysis of our patient archiving system (IS-H SAP, Siemens AG Health Care Sector, Erlangen, Germany) identified all patients which were treated due to a femur fracture in our hospital (university maximum care provider) from January 1, 2010 to December 31, 2016 (1,468 patients). Subsequently, all patients with a periprosthetic femoral fracture were determined (178 patients). After exclusion of patients with PPF besides total knee arthroplasty, 93 patients with PPF and an existing THA were included. Due to a conservative therapy, 13 patients were excluded, too. The remaining 80 patients were divided into two groups. Group I represents the osteosynthetic treatment to preserve the implanted THA. These were 42 patients who had been treated with plate osteosynthesis with or without additional cerclages or isolated with cerclages. Group II with 38 patients includes those people who were treated by a change of their endoprosthesis with or without additional osteosynthesis.

The selection decision of the surgical procedure will be determined on the basis of the fracture classification (Vancouver and Johannsen) and in particular on the stability as well as the anchoring type of the endoprosthesis, the comminution zone and the fracture localization [15], [16]. Furthermore, the presence of secondary diseases, especially osteoporosis, previous immobility and multimorbidity were implied. Ultimately, all cases were discussed in our indication meeting and the operative procedure was determined.

Angular-stable non-contact bridging (NCB) plates from Zimmer Biomet Holdings (Warsaw, IN, USA) and angle-stable less invasive stabilization system (LISS) plates from DepuySynthes (West Chester, PA) were used for osteosynthesis. As cerclages 1.5 mm wire cerclages from the company DepuySynthes (West Chester, PA, USA) were utilized. The treatment during endoprosthesis change varied and was adapted to the individual circumstances. In doing so, implants from the following companies were applied:

- Zimmer Biomet Holdings, Warsaw, IN, USA: 12;
- Peter Brehm Chirurgie-Mechanik e.K., Weisendorf, Germany: 8;
- Orthodynamics GmbH, Lübeck, Germany: 7;
- AQ Implants GmbH, Ahrensburg, Germany (Eska Implants GmbH & Co. included): 4;
- Mathys AG, Bettlach, Switzerland: 3;
- DepuySynthes, West Chester, PA, USA: 3;
- AlloPlus GmbH, Saarbrücken, Germany: 1.

Moreover, all patients got the same standard treatment as well as a follow-up according to uniform guidelines.

In all patients, specific characteristics were recorded and compared. They included gender, age at fracture, interval between fracture and implantation, length of in-patient stay, body mass index and osteoporosis (absolute (percentage)). In order to objectify the bone quality, the ratio of medullary canal to cortical thickness was determined on the basis of the corticomedullary index introduced by Barnett and Nordin (BNI) in 1960 on the preoperative x-ray of the pelvis and the anterior-posterior image of the femur, respectively. Additionally, the electronic files and x-rays of all patients were retrospectively evaluated and complications such as infections, re-fracture, loosening, material failure or other complications (hematoma, wound healing disorder, nerve damage) and the time to complication recorded. Furthermore, all patients were re-examined in July 2017 by means of a questionnaire. Thus, complications (see above) were gathered once again and the function of the THA was inquired by means of the score according to Merle d’Aubigné and Postel [17], [18]. This score includes three sections: pain (0–6), agility (0–6) and walking ability (0–6). Pain and agility add up to a functional score with a maximum of 12 points while all sections result in a total score of up to 18 points.

The statistical analysis of the data was done using Microsoft Excel (2013, Redmond, USA) and IBM SPSS software (24.0, IL, USA). The Kolmogorov-Smirnov test and the Shapiro-Wilk test were used to determine the normal distribution of the data. In addition, the Mann-Whitney nonparametric test and the Chi-square test were applied. P values of 0.05 or less were considered as statistically significant.

## Results

In group I the mean follow-up time was 48.5±23 months (4 years) whereas group II amounted 32.5±24.5 months (2.7 years) (p=0.029). The patient’s specifics are shown in Table 1 (Tab. 1). There were no significant differences concerning the interval between fracture and implantation, gender distribution, body mass index and bone quality with regard to osteoporosis and the Barnett-Nordin-Index (BNI). Only the attribute age shows that the group with prosthesis preservation is significantly older than the group with THA change (81±11 years vs. 76±10 years, p=0.047). According to the length of in-patient stay significantly shorter periods were found in the group of osteosysnthesis by means of endoprosthesis preservation (14.5±8.6 vs. 18.0±16.7 days, p=0.014).

The classification of fractures showed a significant difference between the groups. Using the Johannsen classification, a balanced distribution between the three given types was found in group I, whereas in group II an imbalance in favor of Johannsen type II with 71% was given (see Table 2 (Tab. 2)). With regard to the Vancouver classification, type B2 (loose endoprostheses) fractures with 79% and type B3 (bad bone quality) fractures with 5.3% were found only in group II. Besides, Vancouver type B1 (solid endoprostheses) fractures received an osteosynthesis by means of prosthesis preservation (48%) more frequently than a prosthesis change (8%) (see Table 2 (Tab. 2)). Furthermore, significantly more patients, who had a complication, were found in group II (9 patients (21%) vs. 15 patients (39%), p<0.017) (Table 3 (Tab. 3)). Here, in particular, infections but also loosening and re-fractures could be observed (Table 3 (Tab. 3)).

The evaluation of the collected scores is shown in Table 4 (Tab. 4). According to the score of Merle d’Aubigné and Postel, there were significantly better values for the pain in group II with comparable values for mobility and walking ability.

## Discussion

Periprosthetic femoral fractures after hip endoprosthesis are a complex and clinical challenging task. The development of the most effective treatment strategy should be based on the fracture morphology, the type of endoprosthesis with its anchorage and stability, as well as the individual patient’s characteristics [19]. Furthermore, the experience and skills of the surgeon play a crucial role which also have to be taken into account. A successful therapy essentially depends on a correct indication for the selection of the most suitable surgical procedure. According to this, classifications such as the Vancouver or Johannsen classification, from which therapeutic consequences can be derived, are helpful [16], [20]. A general rule states that an internal stabilization should be sought in case of a fixed prostheses without extensive comminuted areas, whereas loose stems always require an additional change of the endoprosthesis [19], [21]. With more than 80% Vancouver type B2 (loose endoprostheses) fractures and type B3 (poor bone quality) fractures were found exclusively in group II with present THA replacement, as expected. Moreover, Vancouver type B1 fractures with solid THA were treated more frequently with osteosynthesis by means of prosthesis preservation (see Table 3 (Tab. 3)). The literature also confirms the good results of the type B1 fracture treatment employing plate osteosynthesis [22], [23]. Whereas in the case of type B2 and B3 fractures, a replacement operation with or without additional osteosynthesis is described as unavoidable [24]. Moazen’s biomechanical analysis has compared the treatment with B1 and B2 fixations. The results show that type B1 fractures can be treated with a single locking plate without complications, whereby a relief should occur. In the case of B2 fractures, a long stem revision with fracture bridging of at least two femoral diameters is recommended. In view of the risk of single plate failure, a long stem revision could be considered for all smashed type B1 and B2 fractures [3]. All these partly dogmatic recommendations must always be weighed in individual clinical cases in everyday clinical situations and should only serve as orientation. An additional use of cerclages for plate fixation is also controversial. Graham describes in his work that the inclusion of wires damages the screw fixings of the plates and that they do not support structural stability. Additionally, they negatively affect the bending properties of the fixation [25]. These results are also proved by Griffiths et al. [26]. A disorder of the periosteal bone healing by cerclages is described as well [27]. However, their targeted use in order to fix fragments in combination with angle-stable plates or during stem revisions is entirely reasonable [20]. Though, in our survey, we found no differences in the groups depending on the cerclages which were used. Taking both groups into account, additive or isolated cerclages were used in more than half of the cases (23/42, 55% vs. 25/38, 66%). In terms of care strategy, the knowledge of the presence of osteoporosis or other bone metabolic disorders such as local malignancies, which may affect the fixation of osteosynthetic materials or healing, is also essential [7], [8]. In particular, the treatment of patients with osteoporosis is challenging. Biomechanical analyzes have shown that a combination of angle-stable, non-angle-stable screws and supplementary wire fixation is sensible [28], [29]. Particularly in the case of partially loosened stems, a problematic anchoring situation can arise when an additional replacement of the endoprosthesis with additive plate osteosynthesis takes place. This can be complicated by cemented stems as well [30]. In our survey, there were no differences in the BNI (corticomedullary index) as a measure of bone quality between the two groups. However, significantly more patients with osteoporosis were found in the group of prosthesis change than in the group with prosthesis preservation (group I: 29% vs. group II: 40%, p=0.021). This fact confirms that in cases with osteoporosis more often no isolated osteosynthesis with endoprosthesis preservation is possible and a change of the prosthesis must be done. In individual cases, these patients with large debris need a proximal femoral replacement [11], [15]. Another crucial factor is the patient’s age at fracture. Thus, our study showed that the patients who had undergone a change of prosthesis were significantly older (group I: 76±10 years vs. group II: 81±11 years, p=0.009). This demonstrates that with increasing age and thus with increasing secondary diagnoses a prosthesis-preserving treatment is difficult to realize. Also Zhu could show this in a meta-analysis for patients on age over 80 years [31]. Regarding this, it was conspicuous that there was no difference in implant life. However, it was found that patients receiving endoprosthesis preservation were released from the hospital significantly faster and had significantly fewer complications. Probably, this can be traced back to the lesser trauma caused by the operation [7], [19]. In summary, the treatment of periprosthetic femoral fractures has a high complication rate and a large number of re-operations. In 71 consecutive patients, Zuurmond found 34 patients (48%) that suffered from a complication, leading to a re-operation in 22 cases (33%). Also in Holder et al. 14 of 45 (31%) patients experienced complications: 6 had deep infections, 6 had nonunions and 2 had aseptic femoral loosening. 11 of the 14 complications were treated with reoperation [32]. Nevertheless, patients with prosthesis change show significantly better scores according to Merle d’Aubigné and Postel in terms of function as well as pain during their follow-up. Here, it should be noted that the follow-up period of the group receiving prosthesis change was significantly shorter than that in the osteosynthesis group (group I: 48.5±23 months (4 years) vs. group II: 32.5±24.5 (2.7 years), p=0.029). This could cause that the values of group I are worse than these of group II. Ultimately, it could also be explained by the better restoration of joint function in contrast to the processes during osteosynthesis.

## Conclusion

The treatment of periprosthetic proximal fractures of the femur is dependent on the classification (Vancouver and Johannsen) and in particular on the prosthetic anchoring as well as the extent of the comminution zone. Older patients and patients with osteoporosis are more frequently treated with an endoprosthesis revision. Patients, who have been treated with an osteosynthesis for preserving their endoprosthesis, showed a shorter length of in-patient stay and fewer complications than people with a replacement surgery. In contrast to that, patients with prosthesis revision had better outcomes concerning the score of Merle d’Aubigné and Postel.

## Limitations

The inhomogeneous treatment by various prosthesis and osteosynthesis systems represents a limitation of this study that is based on the inhomogeneity of the fractures, typical for these types of patients. Further limitations are the retrospective study design, the varying lengths of the follow-up period and the small collective size.

## Abbreviations

- BNI: Barnett-Nordin index
- LISS: less invasive stabilization system
- NCB: non-contact bridging
- NIS: nationwide inpatient sample
- PPF: periprosthetic fractures
- THA: total hip arthroplasty

## Notes

### Authors’ contributions 

DZ und CP contributed equally to this work.

JF initiated the work and is the head of the expert team. CP has carried out the data collection and presentation. She also has contributed significantly to the preparation of the manuscript. DZ was part of the expert team and a major contributor in writing the manuscript. ME as well as RM gave statistical support, endorsed the drafting of the article and revised it critically. CP helped with data collection. TP, AR and JF were mainly responsible for the treatment of the patients as well as members of the expert group. All authors read and approved the final manuscript.

### Competing interests

The authors declare that they have no competing interests.

### Acknowledgements

We acknowledge the support of the German Research Foundation (DFG) and the University Hospital Leipzig within the program of Open Access Publishing. 

### Funding

This study was funded by the German Research Foundation (DFG) and the University Hospital Leipzig within the program of Open Access Publishing. The funding body had no impact on the design of the study, collection, analysis and interpretation of data as well as writing the manuscript.

### Availability of data and materials

The datasets used and/or analyzed during the study are available from the corresponding author on reasonable request.

### Ethics approval

The ethics committee of the University Hospital Leipzig, Germany, granted ethical approval (ref. no. 044/ 14032016). The committee is listed in the Institutional Review Board (IRB) of the Office for Human Research Protections (OHRP) IORG0001320, IRB00001750. All patients of our study gave their written consent for participation and publication of their anonymized data.

## Figures and Tables

**Table 1:**
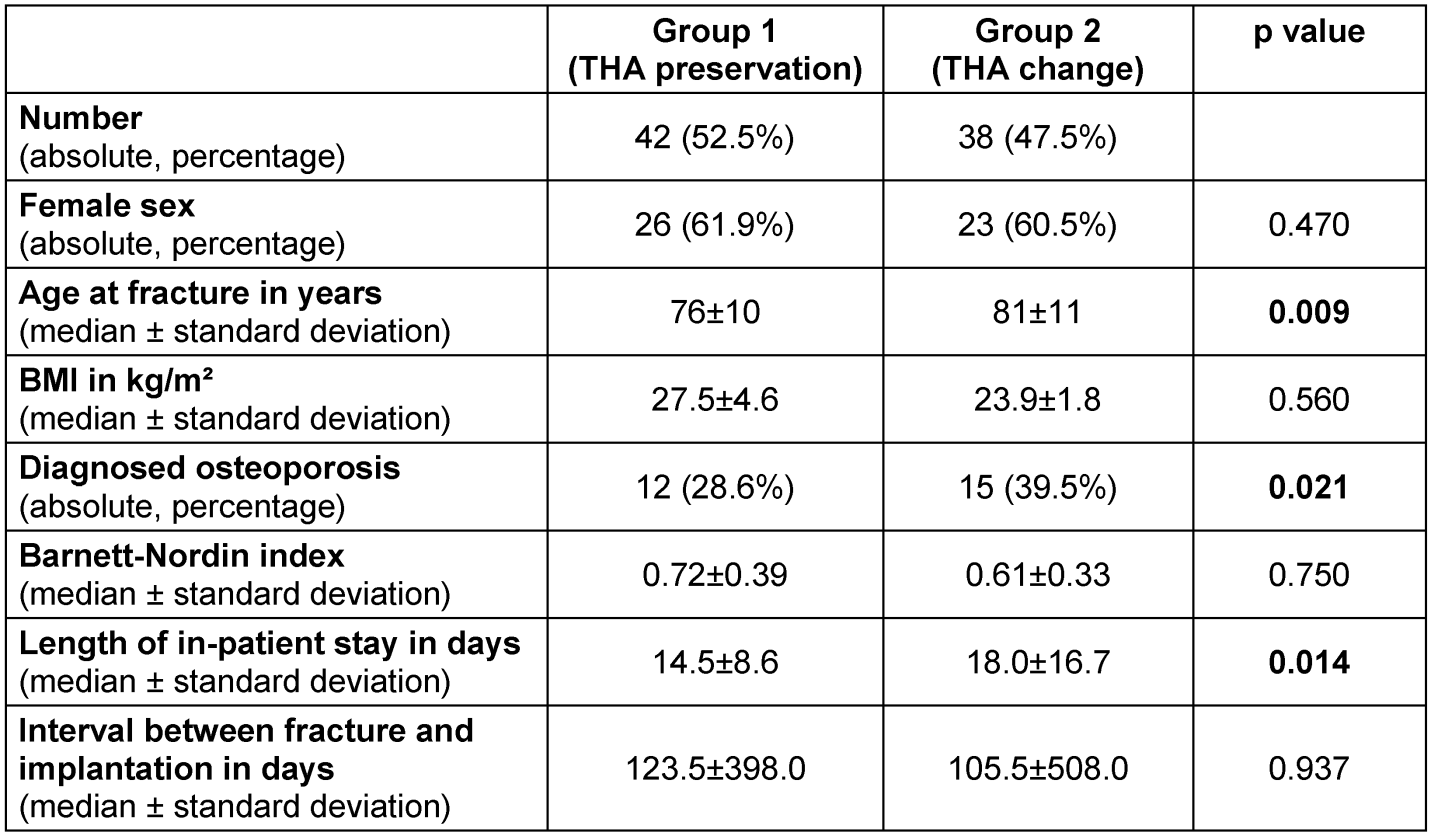
Patient specifics of the two groups with number, female sex, age at fracture, BMI, diagnosed osteoporosis, Barnett-Nordin index, length of in-patient stay, interval between fracture and implantation

**Table 2:**
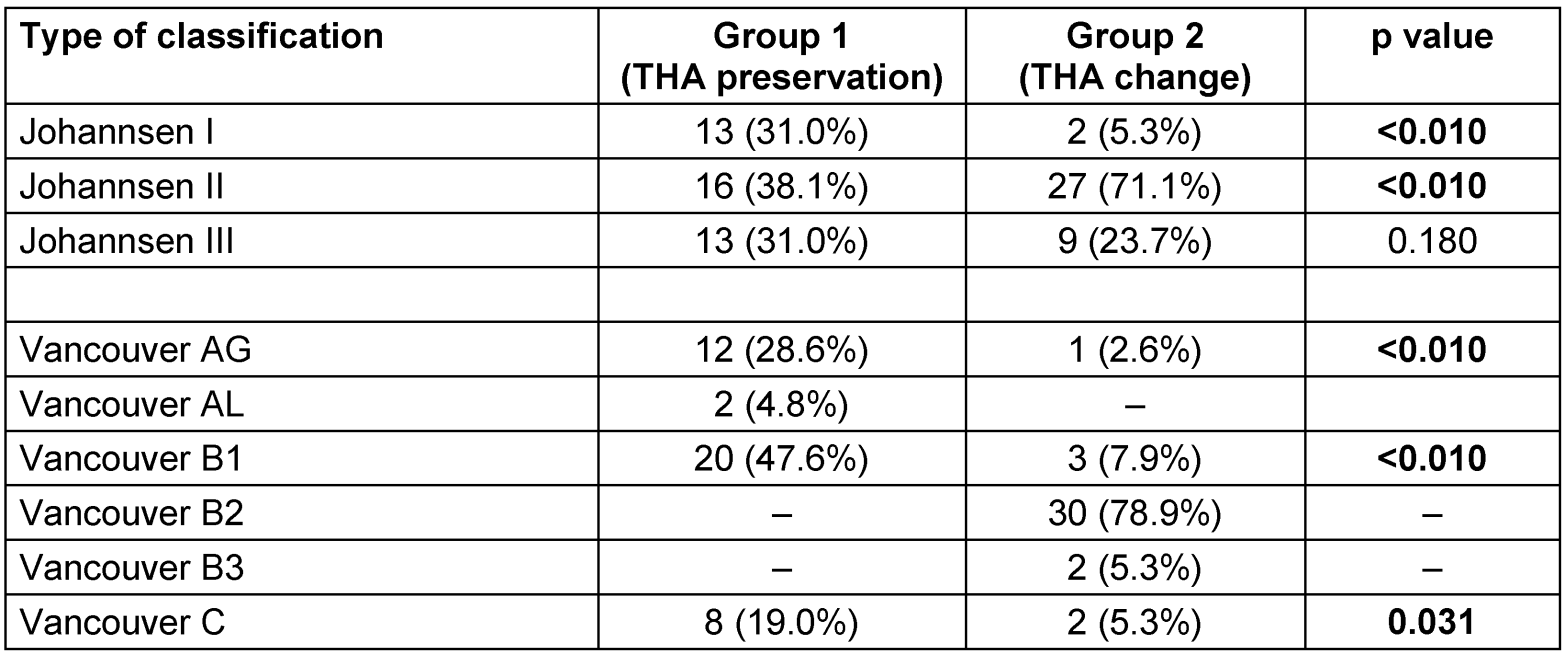
Type of fracture in the two groups according to Johannsen and Vancouver classification

**Table 3:**
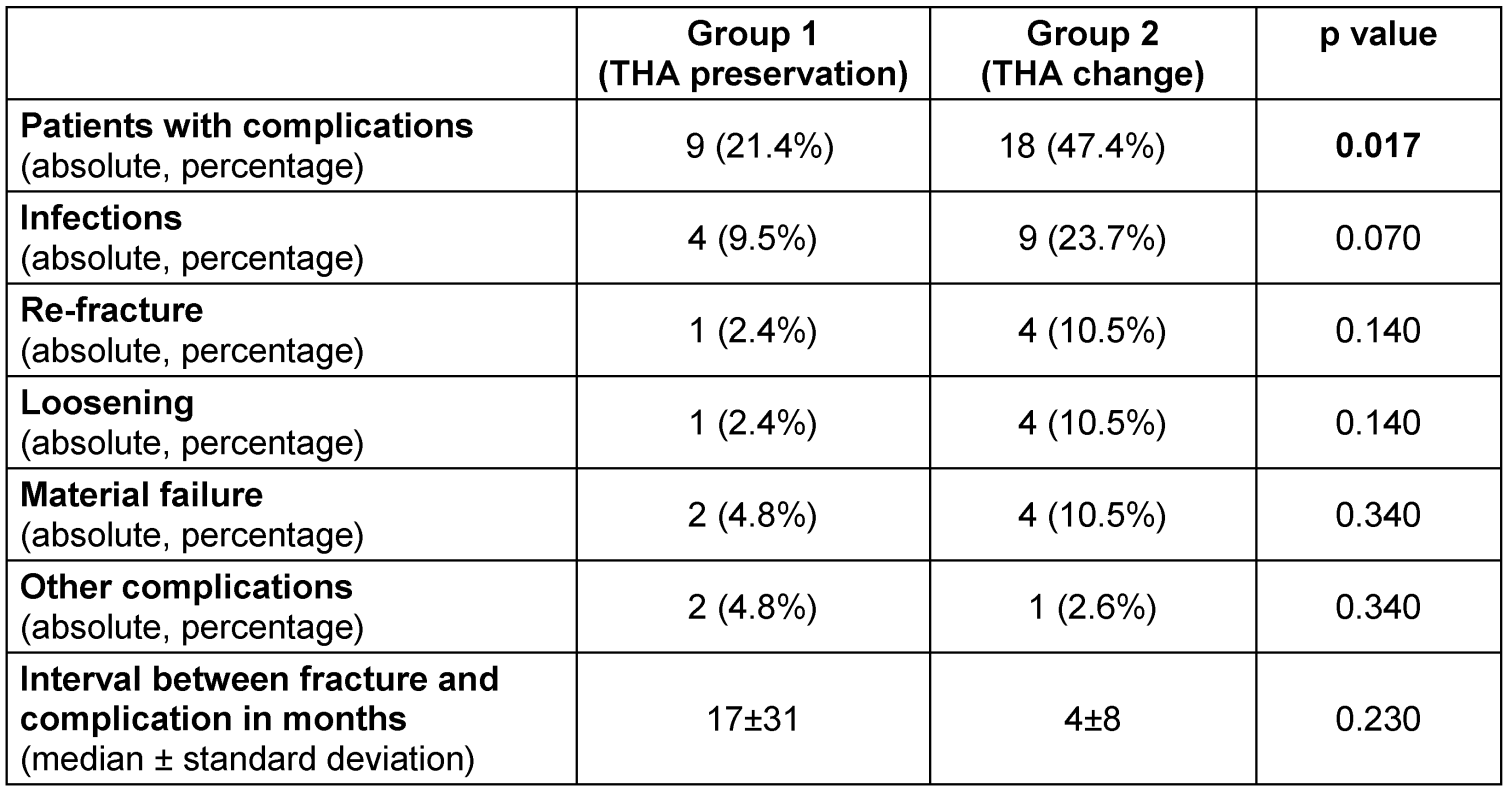
Complications in the two groups with number of patients with complications, infections, re-fracture, loosening, material failure, other complications and interval between fracture and complication

**Table 4:**
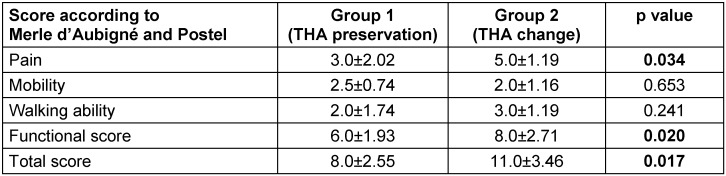
Score according to Merle d’Aubigné and Postel in the two groups (pain, mobility, walking ability, functional score, total score)
